# Advancing the central role of non-model biorepositories in predictive modeling of emerging pathogens

**DOI:** 10.1371/journal.ppat.1011410

**Published:** 2023-06-15

**Authors:** Jocelyn P. Colella, Marlon E. Cobos, Irene Salinas, Joseph A. Cook, The PICANTE Consortium

**Affiliations:** 1 University of Kansas Biodiversity Institute and Department of Ecology & Evolutionary Biology, Lawrence, Kansas, United States of America; 2 University of New Mexico, Department of Biology, Albuquerque, New Mexico, United States of America; 3 Center for Evolutionary and Theoretical Immunology, University of New Mexico, Albuquerque, New Mexico, United States of America; 4 Museum of Southwestern Biology, University of New Mexico, Albuquerque, New Mexico, United States of America; University of Massachusetts, Worcester, UNITED STATES

The COVID-19 pandemic demonstrated the insufficiency of a reactive approach to emerging zoonotic pathogens. With spillover increasing in frequency as environments change and the human footprint continues to grow, pandemic prevention will require predictive models that can identify (i) potential zoonoses with a high likelihood of emergence and (ii) environmental or other features that may trigger a shift in host, vector, or pathogen baselines associated with emergence and/or spillover. Artificial intelligence (AI), and particularly its machine learning and deep learning branches, holds enormous potential for detecting shifts in large-scale biodiversity and disease datasets (genomic, ecological, geospatial, etc.) [1]. Such algorithms can be trained to identify subtle patterns in large volumes of data to yield insights into complex phenomena for which we have limited knowledge of the true cause (s) or predictor (s), as is the case for emerging infectious diseases.

Accuracy of AI or any other analytical approach, however, is limited by the quality, completeness, types, and biases of input data. Models are often hamstrung by unbalanced data. For example, if there are many host records, but few are infected by a pathogen, there may be insufficient information to identify a pattern. This complexity only increases when considering multiple host species, each of which has distinctive ecological requirements, generation time, life history, and dispersal ability (e.g., [2]). Using incomplete data to build predictive models can increase the number of candidate predictors, unnecessarily increasing computational complexity and processing time. Worse, it can produce overfit models that generalize poorly on new data and, in extreme cases, may increase false signal detection [3]. Thorough exploration of variable associations prior to inclusion in a larger model and transparency regarding the limitations of model extrapolation are critical (e.g., [4,5]).

Most emerging diseases in humans come from non-model organisms; therefore, understanding the ecology and evolution of those host species in the wild will be key to identifying features most informative for disease modeling and risk assessment. We focus on the enormous but relatively untapped potential of non-model host biorepositories and their associated databases to fuel predictive modeling of host–pathogen interactions, emergence risk, and pandemic potential across human–animal–environment interfaces. Non-model biorepositories, in this context, including natural history, agricultural, and other biodiversity collections that preserve biological materials (i.e., samples, specimens) in perpetuity. The metadata associated with the specimens warehoused in non-model biorepositories are served publicly online through museum databases such as Arctos (arctos.database.museum) and Symbiota (symbiota.org), or data aggregators like the Global Biodiversity Information Facility (GBIF, gbif.org), iDigBio (idigbio.org), and VertNet (vertnet.org), some of which are interoperable with other digital data streams (GenBank, MorphoBank, etc.) derived from physical specimens. Through the newly established PICANTE (Pathogen Informatics Center: Analysis, Networking, Translation, and Education) initiative, we advocate for a multidisciplinary and, importantly, proactive approach to pandemic prediction and prevention (e.g., [6]) that emphasizes strategic expansion of non-model biorepositories, particularly in biodiverse countries, to stimulate and refine predictive modeling of emerging diseases.

## Going beyond serology

Serological tests or immunoassays screen blood for the presence of specific antibodies as an indication of historical infection. Such tests are a common tool used in disease surveillance because they are fast and affordable, but non-lethal serological investigations rarely, if ever, archive “voucher” specimens (e.g., preservation of host species and/or their tissues in a permanent biorepository) [7]. Thus, the disconnect between biorepositories and biomedical surveillance has created major gaps in knowledge and, critically, in biorepository sampling infrastructure that now limits our understanding of the ecology and evolution of emerging zoonoses and their wild hosts [8].

Serology requires commercially produced (“secondary”) antibody reagents capable of binding to specific immunoglobulins produced by the host. Yet, even within vertebrates, commercial reagents are often unavailable for non-model host species or are, potentially, less specific. As a result, critical model parameters such as baseline pathogen prevalence, transmission pathways, evidence of pathogenesis, rates of morbidity, occurrence of maternal immunity (i.e., placental, colostral transfer), and rates of recovery (e.g., [9]) remain unknown for most non-model wild hosts. In contrast, preservation of a holistic voucher specimen at the time of sampling would allow preliminary serological results to be revisited or extended at a later date, using more sensitive or updated technologies (e.g., whole-genome sequencing, metagenomics, RNAseq, environmental DNA) to fill information gaps. The date of serological screening is also not equivalent to the date of infection, as infection could have occurred months prior to screening. Thus, seropositivity cannot precisely associate a pathogen with the ecological or environmental conditions recorded at the time of sampling. Instead, models based on seroprevalences must consider the breadth of conditions present during the hosts’ life span, which severely limits resolution into biotic, abiotic, and anthropic factors that may contribute to outbreak or spillover. Again, if voucher tissues are preserved, reverse transcription polymerase chain reaction can be used to identify active infections among seropositive individuals, with sequencing then possible on preserved host tissues to identify strains, probe immune responses, and investigate pathogen evolution in detail.

## Work smarter, start with extension

The World Health Organization’s Blueprint [10] aims to “improve coordination between scientists and global health professionals to accelerate research and development related to emerging diseases” [11]. The Blueprint prioritizes pathogens based on their potential to cause the next global pandemic [12]. In this case, pandemic potential is measured based on information often available through non-model biorepositories, such as insights into human–animal interfaces, the evolutionary potential and geographic range of the pathogen, and existence of previous immunity [11]. The WHO Blueprint could be made more effective by including input from biorepository professionals with expertise in field collection methods, taxonomy and systematics, and state-of-the-art long-term specimen preservation methods [7]. Recent, large-scale investigations have screened >75,000 mammals for viruses, but preserved few to no physical specimens (e.g., [13]), even in cases where hosts could not be identified to species. Such an approach precludes verification of host and pathogen taxonomy and limits future extension of initial work using new tools in the rapidly evolving fields of genomics, immunology, or isotopic chemistry [14] and serves to highlight the enormous potential for biorepositories to synergize with the public health and biomedical communities.

## Leveraging biorepositories for pathogen prediction

To maximize information gained, disease surveillance must be designed with verification, replication, and extension in mind [15]. Holistic collection and specimen vouchering [16,17] involve subsampling and archiving multiple parts of an organism to not only answer an initial question, but to also intentionally catalyze diverse scientific inquiry and facilitate integration across disciplines by tying newly derived information (e.g., genetic sequence data, serology results) back to a physical specimen record. Vouchering a subset or, ideally, all sampled taxa in a non-model biorepository ensures future verification, replication, and extension of research discoveries [15].

Biorepositories and their associated databases are rich, openly accessible sources of physical samples and digital data useful for diagnostic testing (e.g., serology, sequencing), monitoring change through time, and building predictive models of host–pathogen–environment interactions. Such collections are assembled by the scientific community over time, through the cumulative contributions of many researchers, agencies, and laboratories that sample natural systems to together produce a temporally deep, geographically broad, and taxonomically diverse global archive of biodiversity from which we can better understand host–pathogen–environment interactions. To be maximally useful, biodiversity databases associated with non-model biorepositories must be openly available online, machine readable, and standardized (e.g., DarwinCore) to allow data from different sources to be combined to increase sample sizes and, therefore, statistical power [18]. Specimens, including frozen tissue resources or cultures, must be available by loan for use in research and diagnostic testing, conditional on compliance with international regulations, including equitable sharing of benefits with international partners [19].

Most modeling applications require balanced input data, yet biodiversity data streams are rarely balanced with respect to taxonomy, sex, or geography and often do not comprehensively represent the entire range of environmental conditions in which we are trying to make predictions. Examples include larger-bodied animals and species of conservation concern that are generally underrepresented in collections or oversampling of highly accessible areas due to logistical constraints [20]. The gap between traditional disease surveillance efforts and sample archival with biorepositories has exacerbated those biases by targeting specific geographic areas or host species following an outbreak. Thus, when model focus is narrowed to a particular place, time, or species, available information may be drastically reduced (e.g., [21]). Moving forward, vouchering specimens as a regular part of disease surveillance will help fill data gaps, even biases, and build foundational infrastructure for biodiversity and disease-related informatics research.

## Non-model host–pathogen interactions, ecology, and immunology

**Fig 1:**
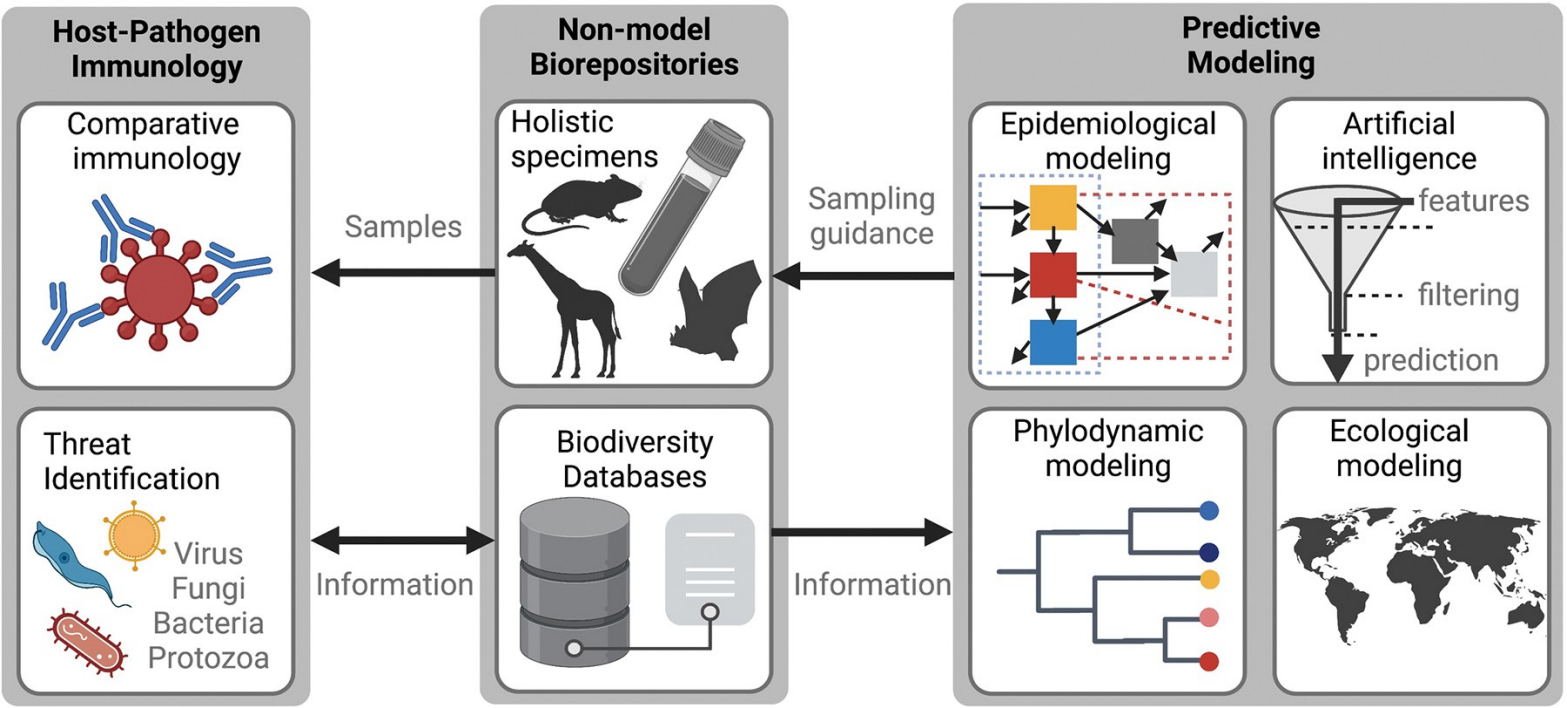
Concept diagram showing an example of how the physical samples and associated data available through non-model biorepositories can fuel diverse aspects of host–pathogen modeling, immunology, and phylodynamics, among other applications relevant to public health. Figure generated with BioRender (biorender.com).

A pathogen can only cause disease when it encounters a susceptible host in an environment conducive to infection. Thus, capturing and understanding disease dynamics in nature requires knowledge of all 3 vertices of the epidemiological triad [22]: pathogens, hosts (plus, vectors), and environments. Before applying models to predict pathogen emergence, it is best practice to first explore the scale (e.g., temporal, geographic, taxonomic), types of variables, and sampling intensity needed to reliably detect deviations from baseline conditions (e.g., [23,24]). Such information can guide strategic, holistic sampling to grow biorepository resources, most critically in biodiverse corners of the globe where pathogen emergence may be more likely [25]. This effort will require funding, expanded biodiversity infrastructure, expertise in holistic field sampling and non-model organism taxonomy, sample archiving with publicly accessible biorepositories, and collaborative, multidisciplinary perspectives (Fig 1). Ultimately, to be proactive, the entire pipeline, from sampling to analysis to policy action, must occur at a rate and scale relevant to public health. Top-down guidance that encourages researchers to contact non-model biorepositories early, as a key partner in disease surveillance, will be critical.

Once infected by a pathogen, transmission potential is determined by the host immune response. The diversity of immune systems on Earth is astounding, yet most immunology and, therefore, investigations of transmission potential have been based on relatively few model species. Pathogens of pandemic potential, however, are mostly hosted by non-model organisms, which have largely unexplored immune systems. The genomic era has brought unparalleled molecular knowledge of such non-model hosts, often illustrating substantial variation even among related species (e.g., [26]). As such, biorepository samples are invaluable resources that can illuminate variation in host susceptibility, transmission potential of wild species, and overall determine the pandemic potential of zoonotic pathogens. For example, the combination of genomes, transcriptomes, and newly developed cell lines [27–29] has propelled our understanding of how bats deal with viral pathogens and why they are common vectors of zoonotic diseases. Yet, this knowledge and equivalent tools are missing for most non-model hosts. Examples include raccoon dogs and Chinese bamboo rats, which have been recently implicated in the origins of the SARS-CoV-2 outbreak in Wuhan Markets [30]. Thus, predicting emergence and potential pandemics is inextricably linked to understanding immunity across a diversity of host species, and, if strategically built, biorepositories can be powerfully leveraged to fill current immunological knowledge gaps.

## Future directions

Pandemic prediction is both challenging and in its infancy. Successful prediction will require proactive multidisciplinary initiatives that intentionally contribute to and expand the sampling and informatic infrastructure of non-model biorepositories. Integrating new computational tools with biorepository archives can be powerfully used to (1) identify new pathogens with zoonotic potential, (2) understand key interactions among pathogens and their wild hosts, and (3) model host–pathogen interactions and risk landscapes in geographic, genomic, and environmental space. Transmission ultimately occurs at interfaces between people, animals, and environments and can be triggered by subtle shifts at any of those levels. We are now tasked to build the primary biodiversity infrastructure necessary to document and assess shifting interfaces and connect these data resources directly to computational pipelines to form an early warning system leading to community-level public health action and policies (DAMA protocol; [6]).

PICANTE is an initiative, centered at the University of New Mexico, which aims to change how the scientific community builds and uses biodiversity infrastructure to proactively identify and respond to zoonotic pathogen emergence. PICANTE is accomplishing this through a series of interdisciplinary collaborations that bridge biological science, engineering, computer science, and the social sciences towards pandemic prediction and prevention by developing affordable, rapid, and scalable screening methods; expanding biodiversity infrastructure and capacities in biodiverse countries; and building predictive models that leverage biodiversity, environmental, and human social and behavioral data to identify and then monitor high-risk interfaces through a global network of biorepositories. PICANTE is distinctive in its proactive approach to emerging diseases—i.e., identifying pathogens and shifts in baseline conditions prior to spillover—and, by design, the project workflow forms a positive feedback loop whereby additional sampling contributes directly to biorepositories, which, in turn, increases input data volumes and improves the accuracy of models derived from biodiversity data streams.
